# Pharmacophore-Based Virtual Screening of Novel Inhibitors and Docking Analysis for CYP51A from *Penicillium italicum*

**DOI:** 10.3390/md15040107

**Published:** 2017-04-05

**Authors:** Yongze Yuan, Rui Han, Qianwen Cao, Jinhui Yu, Jiali Mao, Tingfu Zhang, Shengqiang Wang, Yuhui Niu, Deli Liu

**Affiliations:** Hubei Key Laboratory of Genetic Regulation and Integrative Biology, School of Life Science, Central China Normal University, Wuhan 430079, China; yuan_yongze@163.com (Y.Y.); hanrui11473@163.com (R.H.); caoqianwen615@163.com (Q.C.); j_h_yu@163.com (J.Y.); jiali93@126.com (J.M.); zhangtingfu1@126.com (T.Z.); bananatcm@sina.com.cn (S.W.); 15029756722@163.com (Y.N.)

**Keywords:** *Penicillium italicum*, PiCYP51A, pharmacophore, demethylase inhibitors, virtual screening, molecular docking

## Abstract

Sterol 14α-demethylases from Cytochrome P450 family (CYP51s) are essential enzymes in sterol biosynthesis and well-known as the target of antifungal drugs. The 3D structure of CYP51A from *Penicillium italicum* (PiCYP51A) was constructed through homology modeling based on the crystal structure of human CYP51A (PDB: 3LD6). Molecular dynamics (MD) simulation was operated to relax the initial model and followed by quality assessment using PROCHECK program. On the basis of the docking information on the currently available CYP51s with the patent demethylase inhibitors (DMIs), pharmacophore-based virtual screening combined with docking analysis was performed to pick out twelve new compounds from ZINC database. Six hits revealed in the ligand database suggested potential ability to inhibit PiCYP51A. Compared to patent fungicide triazolone, the top three lead compounds had similar or higher affinity with the target enzyme, and accordingly, exhibited comparable or lower EC_50_ values to *P. italicum* isolates. The results could provide references for de novo antifungal drug design.

## 1. Introduction

Citrus is the world’s most common crop grown in over 100 countries worldwide [1]. The fungal diseases of blue mold on post-harvested citrus fruits, caused by *Penicillium italicum*, have led to 30%–50% loss of annual citrus economy in China [2]. Application of synthetic fungicides is the preferred method to control post-harvest diseases caused by fungal phytopathogens in fruits and vegetables. Sterol 14α-demethylases from Cytochrome P450 family (CYP51s) are essential enzymes in sterol biosynthesis and well-known as the target of antifungal drugs, which was membrane-bound and difficult to solve its crystal structure [3,4]. Demethylase inhibitors (DMIs) have been exploited as azole-antifungal drugs to inhibit lanosterol 14α-demethylase (CYP51), impairing ergosterol/lanosterol metabolisms and finally resulting in fungal growth inhibition [5]. DMI fungicides have been widely used in medicine and agriculture, but the resistance to DMIs has been reported in many fungal species [6,7]. It is imperative to design new and efficient fungicides for the treatment of pathogens of plants based on the CYP51 structure.

**Figure 1:**
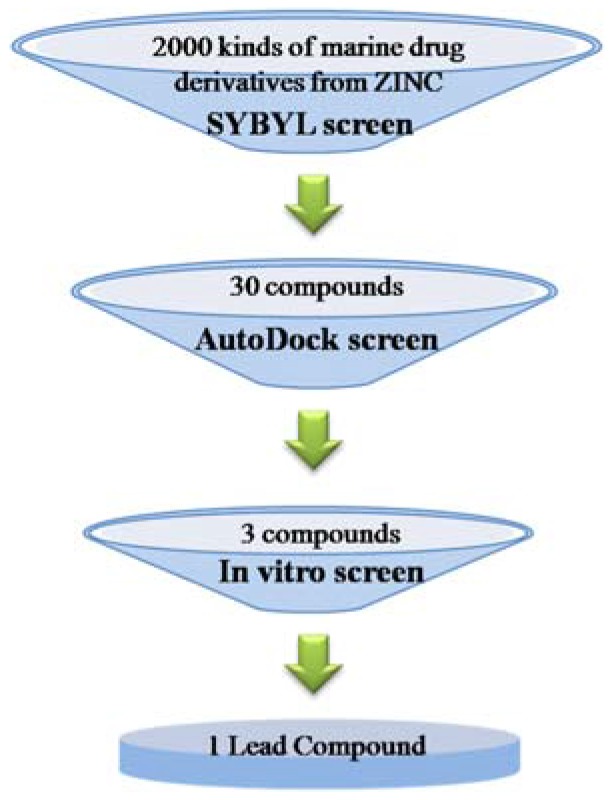
Schematic for the high throughput virtual screening protocol in the present study.

Natural compounds extracted from marine fungi can be used as fungicides for phytopathogenic fungal pathogens [8,9,10]. Based on their structure characteristics, these natural antibiotics from marine fungi can be divided into several typical categories including aminoglycosides, macrolides antibiotics, microlides antibiotics, quinones, peptides, enzymes and heterocyclic [11,12,13]. At present, *Penicillium italicum* has raised increasing concerns due to its serious threat to citrus fruit storage and transportation, and identifying novel drugs against the pathogen is of great significance for the control of blue mold. Currently, the effective fungicides reported in the control of blue mold include imazali-, triazolone- and prochloraz-related chemical drugs, all classified as benzene and nitrogen heterocyclic ring compounds, sharing structural similarities with the marine anti-fungi drugs [14,15]. This makes it possible to find valuable lead compounds by high throughput virtual screening based on the pharmacophores generated from particular marine natural fungicides (Figure 1).

Virtual screening supported by large-scale chemical libraries has been developed to be an important tool in novel lead molecules discovery [16,17]. Ligand-based screening techniques have the advantage of finding lead molecules based on a set of pharmacophore elements derived from a specific functional ligand family [15,18,19,20]. Owing to the membrane-associated characteristics for all the eukaryotic CYP51s, it remains a challenge to solve their crystal structures. Homology modeling has been widely applied in three-dimensional model building of CYP51s to understand molecular interactions between inhibitors and target enzymes [17,21]. The crystal structure of *M. tuberculosis* CYP51 (MtCYP51) has been used in many CYP51 modeling studies [4,22,23]. However, MtCYP51 has only 27.2% sequence identity with the PiCYP51A. The structure of human CYP51A, with 37.7% sequence identity to the PiCYP51A [19,24], is a more suitable template to construct the PiCYP51A structure model in the fungicide screening. In the present study, based on PiCYP51A target protein, we use the natural fungicidal components of marine fungi as reference compounds for the design of novel antifungal agents based on pharmacophores.

In this study, the three-dimensional model of CYP51A from *P. italicum* isolate (HS-1) was constructed based on the crystal structure of human CYP51A (PDB: 3LD6). The protein model further relaxed by molecular dynamics (MD) simulations and evaluated by PROCHECK program was used in pharmacphore-based virtual screening to identify new head antifungal compound(s) from ZINC database (http://zinc.docking.org/).

## 2. Results

### 2.1. Homology Modeling of PiCYP51A

**Figure 2:**
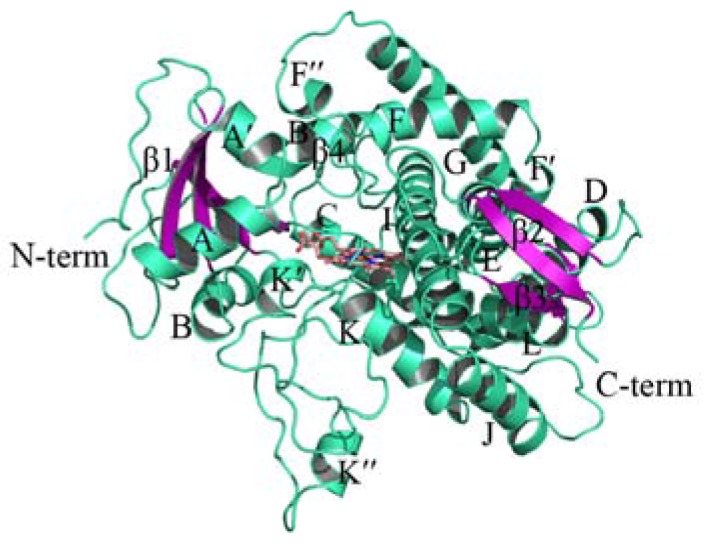
3D model of *Penicillium italicum* (PiCYP51A) shown in ribbon representation.

The human CYP51 has an identity of 37.66% on amino acid sequences with PiCYP51A, and thus is suitable to be the template for the homology modeling in this study. The first 39 residues in the constructed model were truncated due to the lack of N-terminal residues of human CYP51 deposited in the PDB database. The initial model was relaxed by MD simulations to achieve the stable 3D structure of PiCYP51A (Figure 2). The quality of PiCYP51A model was evaluated to be rational with 86.4% in favored core regions, 12.4% in allowed regions, 0.7% in generously allowed regions, and only 0.5% in disallowed regions.

### 2.2. Virtual Screening

**Figure 3:**
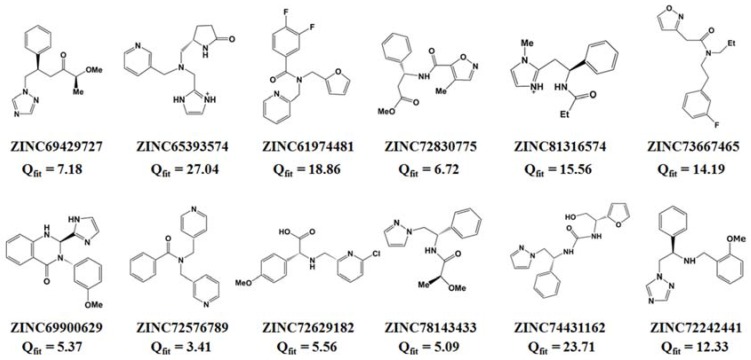
Chemical structures of the twelve compounds retrieved from the ZINC database.

Based on PiCYP51A active site characteristics, virtual screening in ZINC database (about 2000 compounds at beginning) exported the head 30 compounds with desirable query-fit (*Q*_fit_) values. Among the 30 compounds, twelve molecules exhibited similar structure-characteristics to some typical marine antibiotics, as shown in Figure 3. Using *Q*_fit_ 10.0 as cutoff value, six molecules were chosen as candidates in further docking analysis, including ZINC72242441 (*Q*_fit_ 12.33), ZINC73667465 (*Q*_fit_ 14.19), ZINC81316574 (*Q*_fit_ 15.56), ZINC61974481 (*Q*_fit_ 18.86), ZINC74431162 (*Q*_fit_ 23.71), and ZINC65393574 (*Q*_fit_ 27.04).

### 2.3. Molecular Docking with Ligands

**Figure 4:**
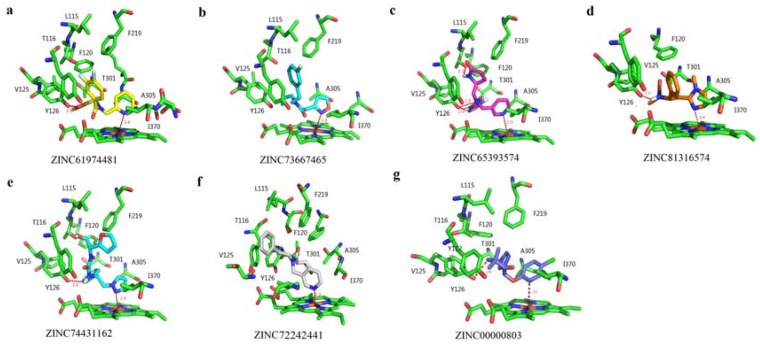
3D-conformation of PiCYP51A docking with six candidate compounds. The six candidate compounds’ IDs are listed as (**a**) ZINC61974481, (**b**) ZINC73667465, (**c**) ZINC65393574, (**d**) ZINC81316574, (**e**) ZINC74431162, (**f**) ZINC72242441, and (**g**) ZINC00000803.

To explore the binding patterns of the active site of PiCYP51A with its ligands, molecular docking analysis was carried out by using FlexX software (University of Hamburg, Center for Bioinformatics, Hamburg, Germany). The docking analysis revealed six ligands of hits with desirable rank scores obtained from FlexX evaluation. The putative conformations of the six candidate compounds in the binding sites of PiCYP51A were represented in Figure 4. The binding energy of the ligands with the receptor was shown in Table 1. According to the binding energy, the top three lead compounds were selected to evaluate fungicidal activities in vitro using binding spectrum and EC_50_ assay.

### 2.4. Assay of Binding Constant (K_d_) and EC_50_

**Figure 5:**
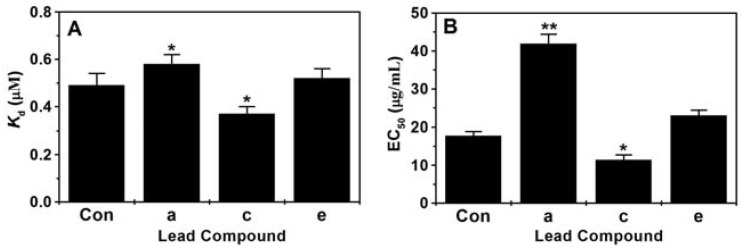
In vitro binding constants (*K*_d_) and EC_50_ of the selected lead compounds. (**A**) In vitro analysis of binding constants (*K*_d_); (**B**) EC_50_ assay; Con: Triazolone, a patent fungicide used as control. (* *p* < 0.05; ** *p* < 0.01).

The binding constants (*K*_d_) of the selected top three lead compounds with PiCYP51A were determined by type II binding spectrum analysis. As shown in Figure 5A, among the four compounds including the patent fungicide triazolone, the lead compound c (ZINC65393574) had the lowest *K*_d_ value, i.e., the highest affinity with PiCYP51A target. In contrast, the other two lead compounds a (ZINC61974481) and e (ZINC74431162) had lower or similar affinity with the target comparing with triazolone (Figure 5A). The inhibitory effects of lead compounds on *P. italicum* isolates were experimentally investigated by EC_50_ assays (Figure 5B). Using the triazolone (EC_50_ 17.74 g/mL) as control, the compound c exhibited significantly higher ability to inhibit HS-1 growth with EC_50_ 11.37 μg/mL, and the compound e had a similar inhibiting effect on the HS-1 growth with EC_50_ 22.96 μg/mL.

## 3. Discussion

For decades, synthetic fungicides based on cytochrome P450 target enzymes have been widely used to control post-harvest diseases caused by fungal phytopathogens [3,4]. Marine drug research has revealed a great number of natural fungicides from marine fungi such as *Ascochyta salicorniae*, *Preussia aurantiaca* and *Penicillium chrysogenum* [8,9,10]. Recently, a set of marine drugs have been reported to have potent activities against *Pyricularia oryzae* in agriculture production [10], which highlights a chance to design novel fungicides based on functional structure similarity. Currently, virtual screening supported by large-scale chemical libraries has been evaluated as a powerful tool to discover novel lead molecules that initiate synthesis of effective antifungal drugs [14,16]. The structure core(s) in the previously reported marine drugs to interfere with certain protein target(s) might provide a good choice of pharmacophore design that could lead a virtual screening to achieve desirable antifungal compound(s). In this study, we report the homolog modeling of CYP51A from *P. italicum* isolate (HS-1) and the further pharmacophore-based virtual screening to identify novel lead antifungal compound(s).

In the current study, we have constructed a PiCYP51A model for the first time with the core region of PiCYP51 proteins (Figure 2), containing the most conserved fold structure reported in the other P450 proteins [16,17,18,21,22,23], including helices E, I, J and L. Each of these helices has at least two aromatic-ring-containing residues (Phe and/or Tyr) involved in the hydrophobic interacting with some specific receptor(s). On the other hand, the present PiCYP51A model also suggested the critical role of nitrogen (N) atoms in the azole and aromatic rings in the coordination bond formation, as experimentally reported for the ligand-heme Fe(II) interaction in PiCYP51A active site [4].

The typical noncovalent bonds interactions have been well documented to contribute to enzyme catalysis, including hydrogen bonds, hydrophobic interaction and van der Waals force [20,25]. All these structure-relating data have been exploited in an efficient pharmacophore-based virtual screening to relax target ligand structure, producing desirable lead compounds with optimal binding energy. The previous reports have described in vitro antibacterial activities of asperflavin ribofuranoside, anthracene glycoside, flavoglaucin and citrinin by certain ligand-target interfering [12]. The similar anti-fungi effects were also reported in the antibiotic activity of diketopiperazine against *Pyricularia oryzae* [13]. In the present pharmacophore-based virtual screening system, the hydrophobic amino acid residues Y112, Y126, F120, F219 and V125 composed a large hydrophobic cleft in the PiCYP51A active site (Figure 4). The aromatic group of the ligands formed hydrophobic interactions with the hydrophobic cleft which fixed the orientation of the inhibitors in the active site [21,26]. The N atoms of the azole ring or hexatomic ring of ligands were bound to heme iron with distances from 2.2 Å to 2.9 Å, respectively (Figure 4). The unique carbonyl (C=O) oxygen atom in compound a, the hydrogen atom attached to amide nitrogen in compound d, and the hydrogen atom attached to N16 (amide nitrogen) in compound e formed H-bond with Y126 about 2.9 Å, 2.0 Å and 2.6 Å, respectively (Figure 4a,d,e). For ligand c, H39 and N1 involved in H-bond interactions with Y126, while another H-bond with a length of 2.1 Å was found between H41 and T116 (Figure 4c). The binding energies of the six ligands were shown in Table 1, indicating the strength of binding interactions of these compounds in the active site. For the selected top three lead compounds a, c and e, the EC_50_ results were well consistent with the *K*_d_ values and also with the binding energy obtained from virtual docking analysis, indicating the effectiveness of the reported pharmacophore-based virtual screening.

## 4. Materials and Methods

### 4.1. Preparation of PiCYP51A Model

The homology model of PiCYP51 was built based on the crystal structure of the human CYP51 (PDB: 3LD6) by SWISS-MODEL, as previously described [22,27]. The model was further relaxed by molecular dynamic (MD) simulations using GROMACS 4.5.5 program package [23,28]. The 5 ns MD simulation was processed under GROMOS96 43a1 force field, within SPC3 water model, and at 300 K/1 atm conditions. The van der Waals and electrostatic interactions were constrained to 0.9 and 1.4 nm reign for the defined long-range interactions. The relaxed 3D model of PiCYP51A was checked by PROCHECK program [24,29]. In addition, the RMSD value between the superimposed model and crystal structure was calculated by Dali sever [25,30].

### 4.2. Virtual Screening

Pharmacophore-based virtual screening was used to find novel potential leads from a ligand database and the general protocol was summarized in Figure 1. DISCOtech module of the SYBYL 7.3 program package [26,31] was used to identify structural features. Further, 3D alignments of the pharmacophore features in desirable molecules with benzene ligand(s) and/or structure core of nitrogen heterocyclic ring, i.e. with the expected structure properties similar to the previously reported marine natural fungicides [10,11,12,13], are found by automatically iterating through experimentally adjustable parameters. The distance tolerance between the pharmacophore features in the overlaid structures was set to 0.25 Å, and gradually increased to 2.5 Å if no model was available with the lower tolerance setting (step size 0.25). The head 30 compounds that matched the main features of the pharmacophore model were exported as candidate hits for following molecular docking. Molecules, structurally matching all the features of ligand model, were selected as hits, and the percentage of the similarity was defined as query fit (*Q*_fit_) value.

### 4.3. Molecular Docking

In order to explore binding patterns of the active sites of PiCYP51A with its ligands, molecular docking analysis was carried out by using FlexX module of SYBYL, a fast and automated docking method. The 3D structures of each compound were built through the SKETCH option in SYBYL with the default setting. Energy minimizations were performed using the Gasteiger–Hückel charge and Tripos force field with a distance-dependent dielectric and the Powell conjugate gradient algorithm with a convergence criterion of 0.05 kcal·mol^−1^. The amino acid residues with atoms located within 9.0 Å distance around the Fe atom in heme group were considered as the essential elements in PiCYP51A active sites. 

### 4.4. Analysis of Binding Constants (K_d_) and EC_50_ for Fungicidal Compounds

Potential effective compounds were purchased from the capable vendors (Enamine Chemical Market), and these commercial lead compounds were exploited to investigate fungicidal activities towards PiCYP51A target. Recombinant PiCYP51A-39, a truncated version lacking N-terminal 39 residues, was heterogeneously expressed in isopropyl-β-d-1-thiogalactopyranoside (IPTG)-induced *Escherichia coli* BL21 (DE3) cells, and the cell lysates containing soluble PiCYP51A were subjected to carbon monoxide (CO) differential spectrometry analysis to ensure enzyme activity and further to make *K*_d_ determination for the commercial lead compounds by binding spectra assay, as described by Li et al. [32]. EC_50_ (the effective concentration to reduce growth by 50%) for each compound was evaluated according to previously reported method [33]. Three replicates were used for each EC_50_ calculation by software SPSS 10.0. Data are expressed as mean ± SE, and differences between means were evaluated by one-way analysis of variance (ANOVA) test.

## Figures and Tables

**Table 1:** The molecular binding energy of PiCYP51A with candidate compounds.

ID	Compound	Binding Energy (kcal·mol^−1^)
ZINC61974481	a	−7.01
ZINC73667465	b	−6.91
ZINC65393574	c	−7.96
ZINC81316574	d	−6.95
ZINC74431162	e	−7.23
ZINC72242441	f	−6.44
ZINC00000803	Triazolone	−7.15
